# Comment on Iavicoli et al. Ethics and Occupational Health in the Contemporary World of Work. *Int. J. Environ. Res. Public Health* 2018, *15*, 1713

**DOI:** 10.3390/ijerph15122654

**Published:** 2018-11-26

**Authors:** Ming-Jui Yeh, Hsi-Chen Liu

**Affiliations:** 1 Department of Health Policy and Management, Rollins School of Public Health, Emory University, 1518 Clifton Rd. NE, Atlanta, GA 30322, USA; 2 Institute of Health Policy and Management, College of Public Health, National Taiwan University, No. 17, Xu-Zhou Rd., Taipei 100, Taiwan; d05848010@ntu.edu.tw

In their recent paper, Iavicoli and colleagues provided a narrative review on the historical development of ethics in occupational health and discussed several ethical concerns; they also hinted the “next step” for resolving the ethical challenges that occupational health professionals (OHPs) will encounter [1]. While this paper contributes to providing an overview of the ethical concerns related to occupational health, several issues could be further addressed.

The substantial ethical analysis in this review paper seems to be minimal, if not absent. The authors summarized many articles that are relevant to the development and concerns of ethics in occupational health, but, just as they stated, “the studies about the procedures for addressing ethical issues in occupational health practice are still few” [1]. The paper could have ended about here, for a review paper does not necessarily need to provide a novel ethical analysis or theoretical breakthrough. However, as the authors mentioned in the introduction section, they will offer “some proposals for solutions” [1]. In the discussion section, the authors also suggested that they have proposed “an integrated approach” and identified “drivers and barriers for correct professional ethics” [1]. These two statements similarly appeared in the conclusion section as well, inviting the readers to seek their core analysis or proposal in the text. 

The analysis of “the three types of ethics” seems to be the one that is closest to an “integrated approach” to address ethical challenges. Nevertheless, as presented in their Table 1 [1], the analysis is more of a comprehensive summary or induction of the existing ethical concerns than a specific analytical approach. Moreover, the contents of Table 1 seem not to be derived from the review in their previous sections. Even if they are so, the authors did not clearly indicate or connect them in a logical manner. This confuses the readers. On the other hand, suppose the analysis of the three types of ethics is indeed the integrated approach they intended to propose; they then could benefit the readers by using more space in the text to elucidate the ideas of this approach.

However, the ethical concerns the authors raised are indeed relevant and should be taken notice of by OHPs and policy makers. For example, as the authors rightly put, globalization has a tremendous ethical impact on occupational health, especially on worsening health inequalities among workers in developing countries and migrant workers. Governments and international organizations need to establish more strategies to face this problem [2]. Second, ethical concerns for older workers are not only limited to those brought by new technologies, but also the ones related to the working environment where a job can be sustainable [3]. Workers who are exposed to ergonomic risks associated with musculoskeletal problems, older workers, and workers with a lower social–economic status are especially susceptible to this issue. Third, some jobs may disappear in the foreseeable future due to new technologies [4]. While the net outcome of the innovations to human society might be promising, some types of workers are vulnerable to these changes and hence burdened with disproportionate risks [5]. This is also an ethical challenge that should be taken into consideration.

The authors rightly called for a broader attention to the ethical issues in occupational health. For beyond scientific research and technical trainings, OHPs need to be equipped with the analytical skills to deal with the ethical challenges of everyday practices. More substantial ethical analyses would be useful for OHPs, occupational health researchers, and policy makers. 
